# State-of-the-Art Preclinical Photoacoustic Imaging in Oncology: Recent Advances in Cancer Theranostics

**DOI:** 10.1155/2019/5080267

**Published:** 2019-04-30

**Authors:** Sara Gargiulo, Sandra Albanese, Marcello Mancini

**Affiliations:** Institute of Biostructure and Bioimaging of National Council of Research, Naples 80145, Italy

## Abstract

The optical imaging plays an increasing role in preclinical studies, particularly in cancer biology. The combined ultrasound and optical imaging, named photoacoustic imaging (PAI), is an emerging hybrid technique for real-time molecular imaging in preclinical research and recently expanding into clinical setting. PAI can be performed using endogenous contrast, particularly from oxygenated and deoxygenated hemoglobin and melanin, or exogenous contrast agents, sometimes targeted for specific biomarkers, providing comprehensive morphofunctional and molecular information on tumor microenvironment. Overall, PAI has revealed notable opportunities to improve knowledge on tumor pathophysiology and on the biological mechanisms underlying therapy. The aim of this review is to introduce the principles of PAI and to provide a brief overview of current PAI applications in preclinical research, highlighting also on recent advances in clinical translation for cancer diagnosis, staging, and therapy.

## 1. Introduction

The field of cancer medicine requires robust approaches for early detection, characterization, and monitoring of tumors. The noninvasive study of key biological parameters in animal models represents a well-established tool for translation of positive results from biomedical research to the bedside. Imaging techniques provide a valuable contribution to improve earlier diagnosis in oncology, as well as for studying angiogenesis and measuring molecular factors implicated in cancer progression and in the response to therapies. Photoacoustic imaging (PAI) has been extensively tested in vivo in preclinical studies during the past decade, in particular on oncological models, allowing to reduce the number of sacrificed animals at multiple time points. PAI performed over multiple wavelengths (spectroscopic imaging) can detect variations in the concentration of tissue components that are hallmarks of cancer compared to the surrounding noncancerous tissues.

Main advantages of PAI include imaging depths up to centimeter and submillimetric resolution, high contrast-to-noise ratios and spectroscopic imaging, real-time acquisition, lack of ionizing radiation, and integration with ultrasound (US) scanners, as well as noninvasive imaging for longitudinal studies, monitoring cancer progression, and drug delivery. Therefore, biomedical community shows considerable interest in translating this methodology into the clinical field. Depending on the biomedical requirement, the major types of PAI systems available can be briefly categorized as microscopy (PAM), endoscopy (PAE), and computed tomography (PACT, focused in this review). PAM and PAE scanners have been mainly used in mouse models of human diseases to image superficial areas and vascular and visceral tissues, respectively, with higher spatial resolution but limited imaging depth compared to PACT systems. Moreover, PACT platforms may provide cross-sectional and/or three-dimensional PAI of living biological structures. Hence, PACT technology appears to be the most promising for PAI clinical implementation. Currently, PAI instrumentation is commercially available only for preclinical studies; few clinical applications are being explored in oncological trials on patients [1]. The physical fundamentals and the major technological implementations of PAI in biomedicine have been summarized in detail by a recent manuscript and therefore are outside our purposes [1]. In this review, the general principles, current preclinical applications, and potential clinical translation of cancer PAI will be primarily described.

## 2. Principles of PAI and Preliminary Clinical Applications

PAI is a hybrid technique based on the photoacoustic effect, a physical phenomenon in which the absorbed electromagnetic energy is converted to acoustic waves. A short pulsed (<10 ns) laser, comprising multiple wavelengths, is used to illuminate biological tissues, inducing ultrasonic waves arising from several tissue constituents.

A typical PA system includes a short pulsed laser source, an US array transducer for signal detection, a component for signal amplification and digitalization, a system for B mode US and PA coregistration, data acquisition, and images representation. The imaging frame rate of the system is usually limited by the laser pulse repetition rate and the time required for multiwavelength data acquisition. In addition, a repeated wide field illumination is limited by the potential tissue damage. Currently, the commercially available US-PA scanners operate at a repetition rate ranging from 5 to 20 Hz [2].

**Figure 1:**
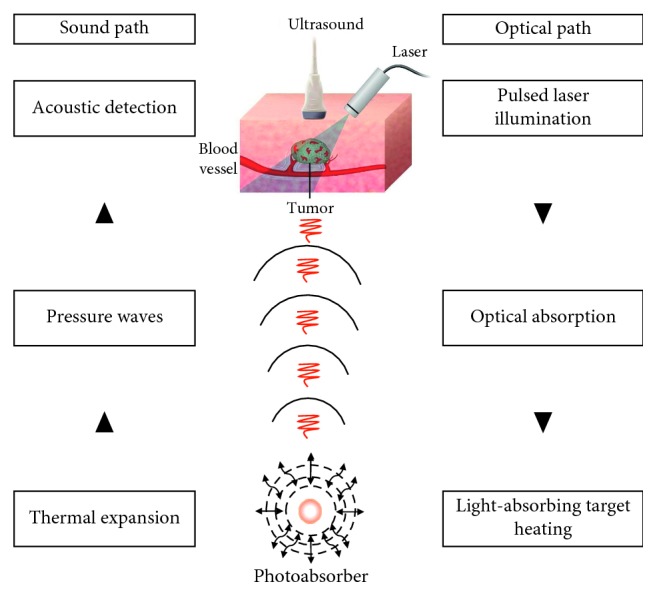
Physical principles of cancer PAI. Short pulsed laser light is used to irradiate the tumor area, inducing ultrasonic waves from endogenous or exogenous photoabsorbers on the basis of thermoelastic expansion. An US transducer is used to detect the PA signal. Contrast obtained from PAI can be useful in characterization and monitoring of tumors (adapted with permission from Valluru and Willmann [2], CC BY-NC-ND license, http://creativecommons.org/licenses/by-nc/3.0/).

The process of PA signal generation can be described in several steps: (1) a target tissue is illuminated by a short pulsed laser; (2) photons propagate unidirectionally into tissues and are absorbed by endogenous or exogenous molecules with optical properties; (3) the absorbed optical energy is partially or completely converted into heat, leading to a transient local temperature rise; (4) the heating induces thermoelastic tissue expansion; (5) tissue thermal expansion changes over time induce local pressure rise, that generates pressure acoustic waves; and (6) broadband acoustic waves are detected by an ultrasound (US) transducer and processed (Figure 1).

Acoustic waves can be produced by employing either a continuous wave laser with intensity modulation at a constant frequency or more commonly a pulsed laser that provides a higher signal-to-noise ratio (SNR). The laser pulse duration is typically ∼10 ns, influencing the fractional volume expansion during the illumination. Approximately each 1 mK temperature rise generates 800 Pa pressure increment that produces a pressure wave ultrasonically detectable. The generated pressure wave includes two components with equal magnitude traveling in opposite directions, as compression followed by rarefaction. Such PA wave propagates through the tissue and is detected by an US multielement 2D matrix transducer array (up to 256 elements) [2, 3].

The image formation is based on two major methods: (1) mechanical scanning using a focused single light beam and a focused single-element ultrasonic transducer (direct method) or (2) using a wide-field light illumination and electronic scanning by a multielement US array transducer (reconstruction method). Each US transducer element receives PA signal over a large acceptance angle, and data are used to reconstruct a tomographic image.

The PA signal is directly proportional to the light absorption by either endogenous or exogenous contrast agents, as well as to the laser light fluence (mJ/cm^2^), generally inducing a range of acoustic frequencies from 1 to 10 MHz [2]. Different tissue components have unique optical scattering and absorption properties for each wavelength; therefore, PAI enables the detection of specific molecules in a complex tissue environment, allowing the study of multiple biomarkers simultaneously. Moreover, the optical absorption coefficient and the amount of each chromophore determine the wavelength and amplitude of the produced acoustic waves. Therefore, the quality and concentration of each optical absorber can be derived through the spectroscopic inversion technique.

The main limitation of optical imaging is the low resolution in deeper tissues, that is related to the physics of light scattering. On the contrary, the acoustic scattering of tissues is approximately one thousand times less than optical scattering. Therefore, compared to pure optical imaging, PAI combines the advantages of higher optical scattering (spatial resolution < 100 *μ*m) with the deeper tissue penetration of acoustic waves (up to 5 cm), with similar sensitivity [2–4].

Approximately, the ratio of imaging depth to the resolution is ∼200, permitting higher resolution imaging across a wide range of imaging depth. Furthermore, PAI shows an absolute sensitivity to relative small variation of optical absorption, hence providing an equivalent percentage change in the PA signal intensity [1].

PAI can potentially improve cancer imaging by combining complementary information and instrumentation and providing morphological, functional, and molecular data in a single image.

The ideal molecular PA probe should have specificity for the biological process under investigation, maximal absorption capability in the NIR window to enable deep tissue imaging, biocompatibility and biosafety, and resistance to photobleaching.

Photons can be absorbed by endogenous or exogenous molecules with various mechanisms, including electronic absorption, vibrational absorption, stimulated Raman absorption, and surface plasmon resonance (SPR) absorption [1].

The PA signal is mainly provided by endogenous molecules, such as hemoglobin (Hb), melanin, lipids, and collagen, or by molecularly targeted exogenous contrast agents, conjugated with antibodies or peptides, displaying optical absorption properties in the near-infrared (NIR) interval (700–1100 nm) [5, 6]. Several biocompatible small molecules (∼1 nm), such as indocyanine Green (ICG), IRDye800CW, Alexa Fluor 750, and methylene blue, show light absorption in this convenient optical window, reducing background signal arising from surrounding tissues. Moreover, gold and silver nanoparticles (NPs) have been widely used as PA contrast agents, due to the SPR effect. The charges on the surface of noble metal NPs, related to oscillations according to the electromagnetic field, result in strong optical absorption properties. Dyes and plasmonic NPs can be also directly conjugated to molecular targeting moieties [6]. In addition, the spectroscopic technique allows to resolve the signal contribution from multiple optical chromophores, thus enabling simultaneous molecular and functional imaging [7] (Table 1). Technological analogies between PA and US imaging systems allow easy implementation of dual-modality scanners, to expand their potential in morphofunctional and molecular imaging [98]. Nowadays, only preclinical PAI scanners are commercially available and have been applied to study the tumor microenvironment, hypoxia, angiogenesis, and metastasis and for targeting specific cancer receptors.

Although PAI appears highly attractive owing to its potential for clinical translatability, it is still facing some operating challenges, and at the moment, a limited number of studies in human patients have been described, as well summarized in recent reviews [2, 99].

On the contrary, the interest in deepening PAI is encouraged by several advantages over other imaging methods currently used in preclinical and clinical fields. Compared to fluorescent tomography, PAI provides deeper penetration and higher spatial resolution across the entire field of view. Beyond functional magnetic resonance imaging (MRI), PAI allows noninvasive oxygenation imaging with high spatial-temporal resolution and relatively low costs. Furthermore, PAI can utilize endogenous contrast agents and it is free from artifacts of acoustic waves as multiple scattering signal interference of returning waves (speeckle) that are found in US imaging. Moreover, PAI can be considered a background noise-free method since nonabsorbing tissue components do not generate a detectable signal, and through proper wavelength selection, it is possible to discriminate the signal generated from endogenous photoabsorbers from the one produced by exogenous probes. Finally, differently from X-ray and PET imaging, PAI does not use ionizing radiations and provides higher spatial resolution. In the last decade, several pilot studies focusing on PAI clinical applications have been underway. A promising research area for clinical PAI is the examination of superficial tissues. The feasibility of breast PAI in patients has been explored as a potential alternative to the standard screening techniques such as X-ray and US mammography, for earlier and more accurate diagnosis and staging. Some prototype clinical PAI mammoscopes have been designed to investigate Hb oxygen saturation and microcalcifications and detect sentinel lymph nodes (SLNs) metastasis of breast tumors with limited data from patients or from bioptic specimens [100–107]. Encouraging results have been reported in differentiating benign and malignant breast masses, with low expense and potentially high sensitivity and specificity, although further technical refinements are needed in the future. In particular, Fakhrejahani and colleagues [105] first performed a pilot clinical study with a prototype PAI machine, in order to compare the distribution pattern of the Hb signal and the oxygen saturation level in primary malignant breast tumors and contralateral normal breasts as control. In this trial, a specific microvascular pattern able to discriminate the normal breast tissue from tumor lesions using PA signal mapping was evident in approximately 75% of patients. Moreover, oxygen saturation values resulted lower in tumors compared to normal contralateral breast tissue, suggesting hypoxia in malignant lesions. In agreement, a significant correlation between histological profile of tumor microvasculature and of hypoxia with qualitative and quantitative analysis of the PA signal was found. Although further technical improvements will be needed, Hb signal mapping has proven promising for breast cancer diagnosis and prognosis, through noninvasive detection of tumor angiogenesis and of oxygenation decrease in malignant lesions. An upgraded prototype PAI system was used on patients by Heijblom et al. [106] to investigate the features of suspected breast lesions compared with X-ray mammography, ultrasonography, and MRI. Using 1064 nm laser light wavelength, the authors demonstrated that PAI can visualize breast malignancies with high contrast and sensitivity and is able to adequately estimate lesion size, showing good colocalization with conventional imaging. However, the presence of high contrast areas around the malignant lesions is indicative for the need of improved specificity, for example, implementing a multispectral technique. In addition, the potential of PAI in detecting skin lesions was based on patients or on biopsies, for example, to differentiate a benign melanocytic nevus from the surrounding blood vessels [108], as well as cutaneous melanoma metastasis in excised SLNs [10]. To date, it is well known that early detection of LNs metastasis is crucial to increase survival in patients with skin melanoma, but sensitivity of conventional cytology, biopsy, and imaging is relatively low. To overcome this issue, multispectral PAI was tested in melanoma patients that underwent lymphadenectomy, using a method with enhanced specificity to differentiate the PA signal arising from Hb and melanin [10]. In accordance with the histological findings, the PA signal from melanin was evident in the upper part of the metastatic SLNs, with different distribution patterns among samples, while it has been not detectable in deeper areas, probably due to the strong melanin optical absorption. Overall, these results appeared encouraging to carry out further studies on the detection of SLNs metastatic foci by PAI, to avoid invasive procedures. In the field of endocrinology, PAI was able to identify malignant and nonmalignant prostate samples based on the deoxygenated hemoglobin (HbR) and lipid signals [109]. A similar analysis was performed on thyroid lesions harvested from cancer patients, and an increase in HbR PA signal intensity was reported in malignant thyroid biopsies compared to benign tissue [110]. Very recently, in vivo combined US-PAI examination has added information to ex vivo studies on human ovaries extracted from patients, highlighting the possibility to discriminate normal ovarian tissue versus malignant lesions through the Hb signal [111–113]. On the contrary, only biopsy samples from cancer of the cervix were examined by PAI, while to date, in vivo studies involving patients are not yet reported [114]. As contrast agents and molecular targeting will become more advanced, the clinical applications of PAI could be improved. Clinical applications above described highlight the relevant potential of PAI translation in biomedical practice to synergically integrate other well-established tools in cancer theranostics.

## 3. Factors Influencing In Vivo PAI in Mouse Models of Human Cancer

Besides technical limits of a given PAI system, several variables should be considered during in vivo preclinical experiments, affecting image quality, inter- and intrasubject reproducibility, and repeatability over time of PA signal quantification.

Animal handling and physiology for a particular mouse strain, age, and gender accounted for an experiment, animal positioning, and anesthesia, as well as operator expertise and standardization of operative procedures were found to be the main sources of variability for in vivo PAI measures.

Precision of PAI quantitative data have been evaluated in living animal models of subcutaneous xenograft tumors, and at the moment, few data are available about total Hb (HbT), HbR, and oxygenated Hb (HbO_2_) measurement [115, 116]. For example, body temperature may affect both peripheral perfusion and cardiorespiratory rates, hence influencing recorded values of endogenous photoabsorbers like HbR and HbO_2_ or the kinetics of exogenous contrast agents in tumors, and therefore, it should be stabilized in the normal mouse range (36–38°C) [115].

Similarly, respiration rate may impact PAI results due to both the effects on oxygenation status and motion artifacts. Therefore, respiration rate in mouse models should be carefully monitored and maintained around 60–70 breaths per minute during PAI imaging [115] to minimize variability, by implementing stable and standardized approaches for anesthetic depth and oxygen supply (21% room air or 100% pure oxygen breathing during general anesthesia). In addition, PA signal variation in subcutaneous xenograft tumors, depending on the positioning of mouse, could be influenced by the pressure on the neoplasia exerted by the animal's body weight and/or animal holder, and/or by the transducer, which in turn may alter tumor shape and hemodynamic. As reported by Costa et al. [116], the shape of a subcutaneous tumor may vary considerably as effect of mouse repositioning, changing the size of an anatomical region of interest (ROI) used for lesion analysis, which in turn can modify the distribution maps of oxygenation and the signal intensity. Therefore, Costa et al. [116] advise to keep the position of the mouse body and of the tumor under examination relatively constant throughout longitudinal studies. Moreover, in serial investigations, it has been recommended that both data acquisition and analysis should be performed using a ROI standardized for size, form, and placement across datasets and by operators with comparable expertise [115]. Anesthesia has been found to be the major experimental source of PA signal variation over time in mouse xenograft models of human cancer [115].

Type and duration of anesthesia influence physiological parameters, such as hearth and breath rate or may induce peripheral vasoconstriction or vasodilation, producing changes of tissue oxygenation, and affect contrast media biodistribution. Therefore, the anesthesia protocol should be conveniently adapted to the study aims, as well as to the imaging technique used [117, 118]. For these reasons, such factors may potentially affect also PA signal assessment in tumors for different contrast agents, but at the moment, only few studies have investigated the influence of anesthesia on PAI. Costa and colleagues [116] have found that Hb and oxygenation PA measures in mouse xenograft models of human cancer significantly change under anesthesia over a period of 75 minutes, so authors suggest to carefully plan in the experimental protocol to image animals at the same time points. Overall, PAI in oncological mouse models should be performed at a defined time after anesthesia induction, to proper inter- and intrasubject comparisons in longitudinal studies, for example, to assess the effects of a new therapy [115]. Moreover, in studies involving contrast agent administration, it is suggested to set standard operating procedures about route and volume of injection and on the biodistribution time.

## 4. Biomedical PAI Applications Based on Endogenous Optical Compounds

Similar to other biomedical imaging techniques, PAI employs endogenous or exogenous contrast agents to enhance soft tissues discrimination for identifying pathological conditions.

**Figure 2:**
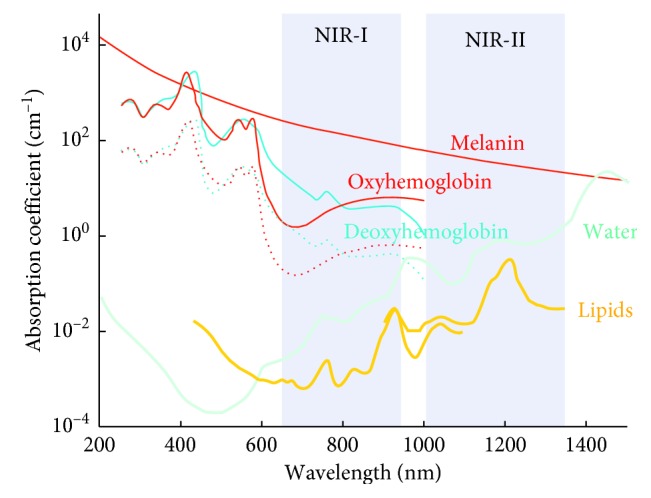
Optical absorption spectra of major endogenous chromophores in tissues of living organisms. The highlighted windows (NIR-I and NIR-II) indicate the wavelength ranges corresponding to minimized optical absorption (reprinted with permission from Deán-Ben et al. [119], CC BY-NC-ND license, https://creativecommons.org/licenses/by/3.0/).

PAI based on endogenous contrast is particularly attractive for oncology applications since it is not necessary to administer exogenous compounds for monitoring tumor progression or treatment response. Hb, melanin, lipids, collagen, elastin, and water are the major endogenous optical absorbers investigated, providing a molecular imaging approach rapidly translatable [119]. Endogenous chromophores have different absorption spectra in the NIR range between ∼700 and 1100 nm, and therefore, specific wavelengths can be selected to obtain maximum contrast in PAI from these compounds. Due to its valuable absorption properties, Hb of red blood cells represents the primary biomarker for a label-free PAI of cancer, allowing the detection of the abnormal vascular network development in tumor growing. Likewise, melanin is capable to generate a strong PA signal, and it has been effectively tested for melanoma detection. Even if different research fields have highlighted the feasibility of endogenous contrast also for lipids, collagen, elastin, and water, they have limited applications for cancer PAI. These biological constituents have shown a lower absorption coefficient than Hb in the NIR region and a signal-to-noise ratio not enough for a reliable contribution to tumor visualization (Figure 2).

### 4.1. Oxygenated and Deoxygenated Hemoglobin

Angiogenesis, referring to the formation of irregular and hyperpermeable blood neovessels within a tumor, is one of the most relevant biomarkers of cancer, which in turn affects the delivery of nutrients and oxygen into neoplasia. Overall, the tumor growth is characterized by a high oxygen consumption rate but at the same time to a decrease of oxygen saturation in the central region. These factors promote uncontrolled tumor cell proliferation, malignant progression, and metastasis, leading to the formation of hypoxic areas associated with chemotherapy (CHT) and radiotherapy (RT) resistance. Using Hb as the endogenous contrast agent, PAI may represent a noninvasive, cheaper, and more accessible way than conventional methods such as oxygen needle electrodes, PET, or BOLD MRI, to provide information about oxygenation status of tumors. Both HbR and HbO_2_ can be used for tissue characterization by in vivo PAI, since in the NIR region, they show distinct absorption peaks at 760 nm and 850 nm, respectively. Taking advantage from this difference in absorption spectra, PAI may provide information about oxygenation status of tumors lesions, enabling the discrimination between benign and malignant lesions, based on a lower HbO_2_ PA signal in many of the most aggressive types of cancer. Nevertheless, PAI has certain limits for determination of blood oxygenation in deeper tissues proportional to the wavelength-dependent optical attenuation [120].

The potential of PAI in oncological screening, focusing on angiogenesis, oxygen saturation, and drug response evaluation, has been examined in different murine xenograft tumor models [121] or in mice with brain neoplasia [7, 8]. Breast cancer, the most frequently diagnosed neoplasia among women worldwide, has been the most widely examined by PAI using xenograft or genetically modified (GEMs) mouse models. Based on oxygen saturation, HbT, and lipid content signals, Wilson and colleagues highlighted the capability of PAI to differentiate the normal mammary gland from hyperplasia, ductal carcinoma in situ, and invasive breast carcinoma in a transgenic mouse model of breast cancer, as confirmed by the histological classification [9]. Spectroscopic PAI revealed a significant increase of the HbO_2_ signal and an analogue decrease of both HbT and lipid content during tumor progression compared to normal breast tissue. The authors hypothesized that the increased oxygen saturation was due to a transition from a prevalently aerobic to anaerobic metabolism, while a low lipid content was related to the replacement of fat tissue by cancerous tissue. The unexpected lower levels of HbT in the most aggressive breast cancer histologies have been confirmed by ex vivo complementary analysis, which assessed a reduction of Hb concentrations in the same mouse model of invasive carcinoma. Interestingly, in this peculiar experimental system, lipids analysis was feasible, showing a distinct absorption peak in the range of 680–900 nm, even if lipids have a markedly lower optical absorption coefficient compared to Hb, probably due to the high proportion of fat in mammary glands. Very recently, breast cancer PAI has achieved effective translation from preclinical research on animal models to the clinical trial, and encouraging results have been described for improving specificity and sensitivity in differentiation of benign and malignant breast masses using a dual modality US-PAI [107]. Morphofunctional features, as well as Hb amount and intratumoral oxygenation status, were assessed in a perspective, multicenter study using a prototype of the hybrid US-PA device and correlated with histopathological findings.

The performance of the US-PAI approach in this study was found favorably correlated with other morphofunctional breast imaging modalities such as nuclear medicine, X-ray mammography or grayscale, Doppler, and contrast enhanced US (CEUS) alone, with the potential advantage of reducing the false-positive diagnosis and biopsies of benign masses. Further improvement of knowledge about US-PAI-derived images analysis and interpretation of quantitative results is required to consolidate its use in clinical practice. Similarly, prostate cancer is an aggressive and commonly diagnosed cancer in men, characterized by high morbidity and mortality rates. Hence, there is a growing interest to develop relatively cheap, low time-consuming approaches with minimal invasiveness for early diagnosis and having improved sensitivity and specificity compared to conventional prostate-specific antigen (PSA) blood test and ultrasonography-guided biopsy. In the last years, the ability of PAI to identify tumoral lesions has been tested both ex vivo and in vivo on canine prostate [122], as well as in a xenograft mouse model of the prostate tumor [123], using the Hb signal to image tumor microenvironment and vascular growth. The coregistration of PA and US images has proven to provide complementary contrast and morphofunctional information. The clinical feasibility of these methodologies needs technical improvements, for example, by implementing advanced US and light-delivering transurethral transducers. More recently, ex vivo multispectral PAI of HbR, HbO_2_, lipid, and water signal was performed on human prostatectomy samples, suggesting the potential to differentiate malignant prostate tissue from benign prostatic hyperplasia (BPH) and normal human prostate tissue, as validated by histopathology. The HbR PA signal was found to be significantly higher, and the lipid signal resulted markedly lower in malignant prostate than normal tissue, while only mean intensity of HbR showed a statistically significant difference between malignant prostate and BPH [124].

The potential utility of endogenous Hb and lipid complementary contrast has been investigated also for the assessment of lymph nodes, given their central role in metastatic dissemination. Ex vivo pilot study on normal human mesenteric lymph nodes highlighted the ability of PAI to reveal vascular structures using the Hb signal and external boundaries through the lipid signal, suggesting a potential application in future for the clinical detection of extranodal metastasis in many types of cancer [125]. Accordingly, in vivo spectroscopic PAI in an orthotopic mouse model of squamous cell carcinoma in the oral cavity demonstrated the feasibility to detect metastatic invasion by monitoring changes in HbO_2_ in lymph nodes.

A good correlation of imaging findings with histologic confirmation of the presence of micrometastases was found [126]. Moreover, PAI of the oxygenation status, in particular hypoxia, is receiving increasing attention for assessing malignant progression and metastasis in clinically relevant orthotopic xenograft mouse models of glioblastoma and lung cancer.

**Figure 3:**
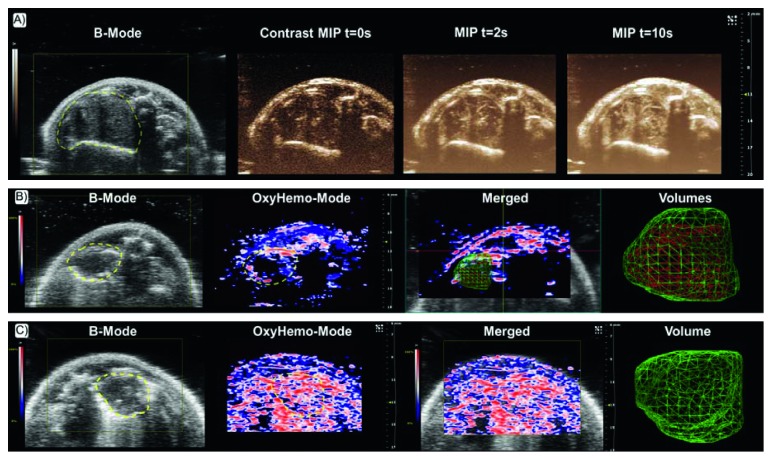
PAI of tumor oxygenation. (a) B mode image of an orthotopic xenograft of lung cancer with corresponding maximum intensity projection (MIP) CEUS images at time points (*t*) 0, 2, and 10 s after microbubbles IV injection. (b) B mode image of a hypoxic lung tumor with corresponding oxygenation map (OxyHemo mode), showing red areas representing the oxygenated part, while blue and dark areas representing hypoxic parts of the tumor. In the following merged US-PA image, the 3D volumes of the whole tumor (green net) and of the hypoxic region of tumor (red net) are reconstructed. (c) Corresponding B mode, OxyHemo mode, merged US PA, and 3D volume images of a more oxygenated lung tumor without hypoxic core (reprinted with permission from Raes et al. [127], CC BY-NC-ND license, http://creativecommons.org/licenses/by-nc-nd/4.0/).

Li et al. [7] demonstrated the feasibility of PAI for noninvasive in vivo imaging of brains of immunocompromised nude mice to detect and characterize intracranial human U-87 glioblastoma xenografts. Functional parameters such as HbO_2_ and HbT of the brain tumors were measured without performing a craniotomy. Mean intensity HbO_2_ value was lower in tumor core compared to surrounding normal brain, suggesting hypoxia development which is correlated to cancer invasiveness, while HbT resulted higher, which is indicative of upregulated metabolism and angiogenesis. This pioneering study aimed to put the basis for an improvement of PAI strategies, useful to accelerate the development of novel molecular anticancer therapies. Target probes at specific molecular markers expressed on the endothelial and tumor cells surface and technical improvements will potentially give a strong contribution to brain PAI applications. Likewise, lung cancer is among the leading causes of cancer-related death, and therefore, more accurate and predictive preclinical protocols to study cancer biology and the response to new anticancer in animal models are needed. PAI, in association with CEUS, showed promising application to investigate progression and to characterize relevant hallmarks such as oxygenation, perfusion status, and vascularization of tumors in a orthotopic mouse model of lung cancer [127] (Figure 3).

In an integrate approach including US, bioluminescence imaging (BLI), and X-ray computed tomography (CT), PAI contributed to add crucial data about tumor hypoxia, a major parameter influencing tumor resistance toward RT and CHT.

A multimodal imaging approach, combining US, BLI, and PAI, was also employed to longitudinally follow tumor growth in an orthotopic mouse model of bladder cancer, successfully mapping the variation over time in oxygen saturation from earlier to more advanced stages of tumor development [128]. A reliable, real time, and comparatively inexpensive imaging strategy using US and PAI is highly desirable in clinic since therapeutic response of bladder cancer can vary widely among patients, and consequently, intensive monitoring is needed to predicting the personal outcome. Overall, assessment of hypoxia levels and microvascular network may be useful in planning and evaluation of the potential response to anticancer therapies. In particular, studies in preclinical models have demonstrated the utility of PAI for monitoring tumor response to antiangiogenic treatments.

It is well known that hypoxia may affect prognosis and CHT and RT efficacy. Rich and Seshadri [129] have described the utility of PAI, noninvasively measuring changes in HbT and HbO_2_ in patient-derived xenograft (PDX) models of head and neck cancer, for assessing tumor following radiation alone or associated with CHT. The authors demonstrated that PAI is able to detect in vivo early changes in oxygenation of tumor as index of responsiveness to RT.

They found that 24 hours after treatment, with both CHT and RT, a variable tumor response was evident, showing from mild changes to significant increase of HbO_2_ measurement compared to baseline. Good correlation was found among PAI and other conventional methods of tumor oxygenation status measurement, like oxygen-enhanced MRI and the percentage of the viable tumor assessed by H&E histology. Moreover, a significant correlation was reported between the percentual variation of HbO_2_ 24 hours after treatment and of tumor volume assessed 2 weeks later by US, suggesting that PAI may aid treatment planning earlier than conventional morphological outcomes. Complementarily, Costa et al. [130] highlighted in the same mouse model the potential utility of in vivo PAI to predict the individual response to RT.

This recent study found that tumors with higher HbO_2_ baseline estimates are better responders to RT compared to ones with inferior oxygenation levels, especially after low radiation dose treatments. In comparison, tumors unresponsive to RT showed a raising trend of HbO_2_ over time. These results suggested that, using HbO_2_ readout, PAI could be a promising tool to successfully guide personalized therapeutic protocols not only by rapidly assessing the effectiveness of conventional anticancer treatments but also by preliminarily predicting the responsiveness of the tumor to RT. Hysi et al. [131] investigated the feasibility of PAI for monitoring changes in the tumor vasculature of an orthotopic mouse model of breast cancer after administration of liposomes containing doxorubicin, showing a significant drop in the HbO_2_ signal early in response of treatment. Their work highlighted the potential of PAI clinical translation for personalized medicine, helping the oncologist to assess therapeutic response rapidly in the single patient.

### 4.2. Endogenous Melanin and Synthetic Melanin-Like Molecules

Melanin is an endogenous pigment, normally presents in hair, nails, skin, and other tissues of living organisms, that is able to produce a PA signal, showing a broadband optical absorption depending on its polymorph structure [11]. Such properties make natural melanin and its synthetic analogues interesting molecules for biomedical applications, like imaging and therapeutic probes [11].

In this view, natural melanin may be potentially useful for PAI by a wide range of wavelength in the NIR interval, and in the last years, there was a growing impulse to the development of melanin-like biopolymers in order to enhance fields of application [11, 12]. Endogenous melanin offers the same advantages previously described for other constitutive chromophores. Moreover, synthetic compounds, such as melanin-like organic NPs, potentially offer high biosafety, promoting the clinical use of contrast agents with low toxicity [11, 12]. Extensive overview on melanin-based contrast agents for PAI has been provided by recent publications [11, 13] and therefore is outside the remit of this paper. Moreover, melanin enables photothermal theranostic approach, causing local hyperthermia after NIR irradiation, to treat several types of solid cancer [11, 12]. In first studies, endogenous melanin has been tested as target for inducing malignant cells death of the heavily pigmented melanotic melanoma. Afterwards, preliminary experiments in mice have demonstrated that melanin-like NPs offer better absorption efficiency and energy conversion to heat after NIR laser irradiation [14].

Given its high invasiveness and metastatic potential, early detection of melanoma is crucial to provide timely an effective therapeutic strategy. Feasibility of PAI based on endogenous melanin has been demonstrated for in vivo evaluation of melanoma both in humans and animal models. In a pilot study, Swearingen et al. [15] applied multispectral PAI of Hb and melanin to improve differential diagnosis of pigmented and vascular cutaneous lesion in patients. The results of this study suggested that PAI could provide complementary information than conventional techniques like dermoscopy, contributing to a better in vivo classification of skin pathologies. More recently, Wang et al. [16] have demonstrated through in vitro and in vivo preclinical experiments that multimodal US-PAI is able to evaluate both the thickness and angiogenesis of cutaneous melanoma, improving diagnosis, prognosis, and noninvasive tumor biopsy. Similarly, SLN biopsy may improve staging and therapeutic management of melanoma. Langhout et al. [10] evaluated the ability of PAI to identify metastasis in lymph nodes excised from patients with cutaneous melanoma. The authors highlighted the sensibility and specificity of the melanin signal to detect cancerous cells, as confirmed by histopathology, encouraging future researches for in vivo clinical applications.

The PA signal of endogenous melanin has been proved to be useful not only in examination of cutaneous melanoma and its nodal metastasis but also to detect melanoma cells in other sites of the body. Recently, Xu et al. [17] demonstrated the possibility to characterize ocular tumors using Hb- and melanin-based PAI both in vivo on the retinoblastoma mouse model and ex vivo in enucleated human eyes bearing uveal melanoma. Another interesting preclinical application showed the PAI ability to noninvasively detect melanoma brain metastases in an orthotopic mouse model [18]. Melanin, HbR, and HbO_2_ content were evaluated by PAI through intact skull using a brain-dedicated bimodal US-PAI probe, showing a clear discrimination of Hb and melanoma cell signal, with higher intensity than the background values assessed in control mice brain. The authors highlighted the possibility to in vivo detect intracranial melanoma and the potential for angiogenesis and hypoxia examination using PAI, aiming at clinical translation in a near future. In recent years, functionalized NPs based on melanin extraction from natural sources or synthetized by chemical oxidation of dopamine (MNPs) have been developed in order to enhance PA contrast and to produce a therapeutic strategy by the photothermal effect [12]. More interestingly, the MNPs are organic and biodegradable and are simply to modify the development of multimodal imaging probes, highly promising for potential clinical translation. Further improvements of MNPs are needed in order to avoid their destruction by the endoplasmic reticulum, to enhance solubility and optical efficiency and to reduce toxicity in vivo [13]. PEGylation has been proven a consolidated strategy to optimize stability, biodisponibility, and safety of MNPs through cell viability and proliferation tests and in mouse model, showing favorable in vivo biodistribution for successful targeting of tumors [12]. The MNPs offer the advantage to both visualize tumors in vivo with high sensitivity and to simplify synthesis of multimodal imaging probes with high theranostic and translational potential. To this aim, Fan et al. [19] developed a biopolymer targeting *α*v*β*3 and performed multimodal PET-MRI-PAI in xenograft mouse models of melanoma. This multifunctional probe allowed to take advantage of PET molecular information, MRI anatomical reference, and high contrast of PAI, improving simultaneously tumor detection and targeting [19]. Very recently, PEGylated melanin-based nanoliposomes for MRI-PAI have been developed to obtain the dual functionality of contrast and therapeutic effect with the same agent. The encapsulation of melanin in PEGylated liposomes increases the phagocytosis of the contrast medium by the tumor cells, enhancing the photothermal conversion of melanin after NIR irradiation and the subsequent tumor cells destruction as demonstrated in breast cancer-bearing mice [20]. Moreover, the melanin nanoliposomes showed improved biosafety in order to make the product easily translatable for theranostic applications [20].

Despite the advantages, PEGylated MNPs have the limitation of lower PA signal strength than inorganic PA contrast agents. To overcome this issue and guarantee at the same time a better biocompatibility, MNPs, modified by citraconic amide surface conjugation, have been developed to induce selective aggregation in a mildly acidic environment, typical of the tumor lesion [21]. This PA contrast agent was tested in a xenograft mouse model of breast cancer, providing a greater and more persistent signal than analogues PEGylated NPs, with improved specific tumor accumulation [21]. Another strategy recently tested to improve biodisponibility, tumor targeting, and photothermal efficacy has been to hide MNPs with red blood cell membrane, resulting in excellent biocompatibility and immune system avoidance [22]. In a xenograft mouse model of lung cancer, such camouflaged MNPs showed excellent photothermal capability, enhanced vascular retention, and improved accumulation at tumor sites.

## 5. Biomedical PAI Applications Based on Exogenous Optical Compounds

The development of exogenous contrast agents is desirable to expand the potential clinical application of PAI, due to their superior absorption coefficients in the NIR spectral region compared to the intrinsic contrasts in biological tissue. Nontargeted PA agents allow to improve tumor detection mainly based on extravasation from the blood stream due to enhanced vascular permeability and retention (EPR) in tumor lesions, while PA contrast media binding a specific biomarker can provide specific information on molecular or cellular processes. Exogenous contrast agents for PAI include mainly organic dyes, fluorescent proteins, metallic or organic NPs, and small molecules suitable for multimodal imaging. The selection of favorable exogenous PA probes for cancer applications is based on the spectral differences of optical absorption compared to endogenous absorbers, their biocompatibility, photostability, and targeting moieties. Few biocompatible dyes have already been approved for clinical use (ICG, Evans blue, and methylene blue), and novel targeted contrast agents for PAI are currently under evaluation in preclinical and clinical settings. Overall, these imaging probes have the purpose to provide theranostic applications for clinical use, with the potential advantages, of circulation time extension, untargeted retention at tumor sites due to the enhanced permeability, specific targeting of tumor cells, reduced toxicity, and capability of simultaneous multimodal imaging and therapeutic activity.

### 5.1. Indocyanine Green

**Figure 4:**
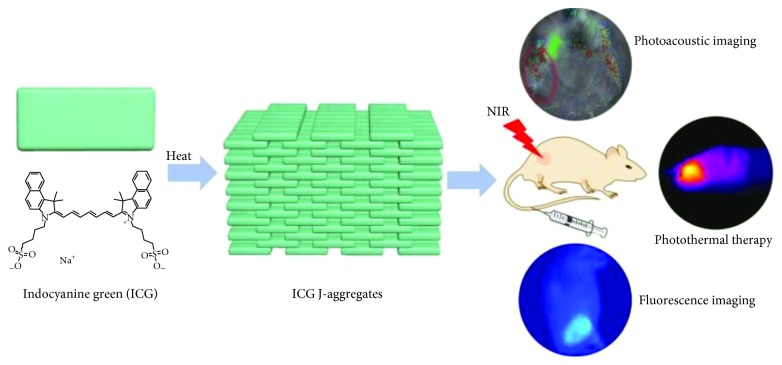
Representative description of the IJA structure and potential use for multimodal imaging and PTT (reprinted with permission from Liu et al. [31], CC BY-NC-ND license, http://creativecommons.org/licenses/by-nc-nd/4.0/).

The indocyanine green (ICG) is a contrast agent approved by US Food and Drug Administration (FDA) for human use that can be used for PAI. ICG absorbs light primarily in the range of 600–900 nm and emits fluorescence (FL) light from 750 to 950 nm [23]. After intravenous (IV) injection, ICG rapidly binds albumin, establishing macromolecules that are unable to permeate the endothelium of many organs. In clinical field, ICG has been used for angiography, liver functional evaluation, and SLNs detection by FL and PA imaging, while in preclinical research, it has been proposed for monitoring tumor growth and therapeutic efficacy [24, 25]. PAI using ICG has been proven to provide early detection of neoangiogenesis in growing neoplasia such as a Lewis lung carcinoma mouse model, based on EPR effect exerted by ICG-albumin macromolecules within the tumor environment [26]. Moreover, this study highlighted that PAI of tumor vascular permeability has the potential to evaluate tumor response to novel antiangiogenic therapies. In addition to diagnostic properties, it has been also proved that 88% of the ICG-absorbed light is converted into heat, and therefore, this feature can be conveniently employed in oncology for photothermal therapy (PTT), leading to effective tumor cell death based on cytotoxicity and reactive oxygen species (ROS) production after repeated irradiations [27]. Shirata and colleagues demonstrated that IV injected ICG was able to accumulate selectively in cells of hepatocellular carcinoma (HCC), among the major primary malignancy of the liver [27]. ICG was able to induce HCC death due to the preserved expression of the uptake transporter for ICG on HCC cells, while function of biliary excretion is altered. In vitro studies showed that the apoptotic effect of ICG after NIR irradiation is essentially mediated by heat and ROS generation, as confirmed in vivo by detection of tumor growth suppression in the Huh7 xenograft mouse model. Although ICG has been used to enhance the PA signal in tumor lesions, this simple contrast agent shows limited applications due to its lacks of specificity, rapid clearance, low photo stability, low water solubility, and tendency to form aggregates [24, 25]. Circulation time and photostability of ICG-based contrast agents have been improved through the encapsulation within NPs, for example, entrapping a high number of dye molecules into bigger NPs using the PEBBLE (photonic explorers for biomedical use by biologically localized embedding) technology. Using this method, Kim and colleagues developed ICG NPs embedded in a biocompatible ormosil matrix [28], while Gupta et al. have encapsulated many ICG molecules into a protein shell derived from the plant-infecting brome mosaic virus (BMV) [29]. Improvement of ICG photostability has been also achieved by liposomal formulation, resulting in enhanced biocompatibility and photothermal efficiency, and in the possibility to surface modification for targeted molecules development [30]. Moreover, liposomal ICG is able to overcome the issue of ICG self-aggregation, which limits the ICG fluorescence intensity and the absorbance ratio. Furthermore, liposomal ICG could potentially combine therapeutic effects based on photothermal heating with chemotherapeutics loading. Theranostic performance of liposomal ICG has been tested in vivo after peritumoral injection in a xenograft mouse model of 4T1 breast cancer. PA signal from tumors resulted a significant decrease after laser irradiation, suggesting that ICG molecules, delivered by the liposomal shell, exerted a successful phototherapeutic effect [30]. In this perspective, liposomal ICG could be promising for clinical translation in oncology due to the use of phospholipid and ICG already approved by FDA and in light of the improvements of imaging and photothermal capability compared to free ICG. Similarly, Liu and colleagues tested in the same breast cancer mouse model a biocompatible nanosized ICG J-type aggregate (IJA), to enhance the in vivo imaging performances and photothermal conversion efficacy of ICG [31]. The ICG-J aggregate is produced starting from the ICG in aqueous solution, and both in vitro and in vivo experiments confirmed that it is simply converted into free ICG after tumor cells retention with high biosafety. Moreover, using this dye assembling preparation method, the drawback of ICG photobleaching was significantly reduced compared to plain ICG, with subsequent improvement of photothermal capability. Furthermore, ICG-J is characterized by a favorable red shift of the absorption peak named “J-band,” and in vivo studies highlighted that its narrow and intense PA peak was able to improve resolution at deeper tissue. Overall, these advantages could be potentially useful for future theranostic applications in oncology [31] (Figure 4).

ICG conjugation with nanomicelles based on the 30k biocompatible hydrophilic polymer polysarcosine (PSar) has been proposed as an alternative strategy to conventional PEGylation, for improving ICG tumor uptake in a faster and higher contrast way [32]. Both in vitro tests and in vivo PAI demonstrated a rapid accumulation of ICG-labeled PSar30k in colon26 tumor cells and bearing mice, leading to higher cancer-blood ratio in comparison to PEGylated ICG. The authors hypothesized that even if ICG-PSar could be mainly retained by the tumor through a passive effect, its cellular uptake could be partially due to micropinocytosis, thus favoring an early in vivo tumor accumulation after injection. Further studies are required to identify the target molecule of PSar in order to select the most suitable cell lines for this agent and to exploit its high contrast capability for guiding tumor resection.

**Figure 5:**
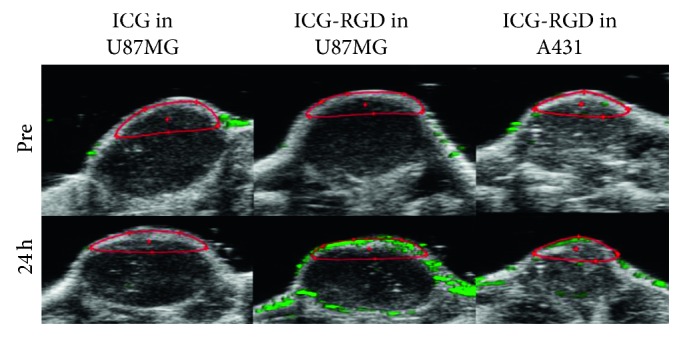
Representative PA images of U-87 MG and A431 xenografts acquired before and 24 hours after injection of ICG alone and ICG-RGD. In contrast to epidermoid carcinoma, the PA signal intensity in glioblastoma was significantly higher after ICG-RGD injection compared to measurements assessed at baseline and after free ICG administration (red region of interest) (adapted with permission from Capozza et al. [24], CC BY-NC-ND license, http://creativecommons.org/licenses/by-nc-nd/4.0/).

Similarly, molybdenum disulfide (MoS_2_) nanosheets (NSs) have recently gained prominence in biomedicine due to their high biocompatibility and capability to load optical dyes on the surface for efficient tumor targeting. MoS_2_-ICG NSs has a wavelength absorption peak at 800 nm, allowing greater penetration depth and sensitivity for in vivo PAI of orthotopic glioma [33]. In vivo PAI revealed MoS_2_-ICG NSs accumulation at the tumor site from 3 to 5 hours after injection, likely due to both EPR effect and albumin receptor-mediated mechanism. Moreover, an imaging depth of 3.5 mm at the tumor site was reached, as demonstrated by MRI cross-sectional images. Overall, MoS_2_-ICG NSs have showed desirable features for in vivo PAI in orthotopic mouse model of deep brain glioma, with potential clinical translation in future. In parallel, Chen and colleagues [34] have reported that molybdenum selenide- (sMoS_2_-) ICG NSs show high photothermal capability and have validated their use for both PAI and PTT in a xenograft mouse model of 4T1 breast cancer, highlighting their potential for theranostic clinical applications. Recent progresses in preclinical multimodal imaging has been reported by Swider and colleagues [25] that developed polymeric NPs of D,L-lactic-co-glycolic acid (PLGA) entrapping perfluoro-15-crown-5-ether (PFCE) imaging agent for ^19^F MRI and ICG, both approved for clinical use. The ICG encapsulated in these NPs results was characterized by increased half-life, slower protein bond, and improved photostability. Moreover, PLGA-PFCE-ICG NPs allow to combine the high sensitivity and fast acquisition of PAI with the greater tissue penetration and quantitative analysis capability of MRI [25]. The authors demonstrated the feasibility to use PLGA-PFCE-ICG NPs for in vitro primary human monocyte-derived dendritic cells (DCs) labeling and to detect in vivo the cellular signal after their intramuscular administration in a mouse model. In addition, they were able to follow labeled DCs in lymph nodes draining the injection site by dual-modality imaging, highlighting the potential for implementing these NPs in cell tracking and cellular therapies. Furthermore, their biochemical features could allow easily modifications for targeting imaging and drug delivery. Therefore, in the last years, ICG has been modified to improve both biochemical properties and molecular imaging capability for theranostic purposes. Last-generation NPs exploit the biocompatibility and desirable optical properties of this ICG dye and have been functionalized with targeting moieties and/or loaded with therapeutic agents [35]. In several preclinical studies, monoclonal antibodies labeled with ICG have been developed for tumor imaging. For example, specific binding of ICG PEBBLEs conjugated to HER-2 antibodies was tested on breast and prostatic cancer cells, highlighting their potential for future in vivo applications in cancer detection and treatment [28]. In following investigations, ICG NPs with improved imaging performance have been tested in preclinical experimental systems. Sano et al. [36] have tested the FDA approved panitumumab (Pan), a antiepidermal growth factor receptor (EGFR) monoclonal antibody, labeled with ICG derivative (ICG-EG4), as a PAI probe to detect squamous cell carcinoma lesions in a xenograft mouse model. In vitro, ICG-labeled Pan has been applied as a fluorescent probe targeting the EGFR, a relevant biomarker of tumor cells proliferation, angiogenesis, and invasiveness. The authors demonstrated by in vivo PA imaging and inhibition studies that Pan-EG4-ICG specifically targets EGFR-positive A431 cells, and therefore, it could be potentially useful to discriminate metastatic tumors and guide therapeutic approach. However, some issues related to the injected dose of ICG-labeled monoclonal antibodies, required to achieve adequate PAI sensitivity, have to be overcome for a safe clinical application. More recently, Wilson et al. [37] have identified the B7-H3 receptor, a potential target distinguishing tumoral lesions from normal mammary tissue, on both the endothelial and epithelial cells in human samples of several subtypes of malignant breast cancer. Bimodal US-PAI with ICG conjugated to a specific antibody against this biomarker was performed in a relevant transgenic mouse model of breast carcinoma. After binding to their molecular target, these antibody-ICG conjugates undergo spectral shifts of optical absorption due to cellular internalization and cleavage of antibody-ICG bond, with specific accumulation in breast cancer lesions. The authors suggested that PAI with this targeted contrast agent could be complementary to conventional mammography and ultrasonography to improve accuracy in diagnosis and staging of breast cancer. Moreover, the same authors have demonstrated in a further preclinical study the potential utility of this B7-H3-ICG agent to intraoperatively guide breast tumors resection, by discriminating normal and pathological mammary gland tissue with high sensitivity and specificity [38]. Another attractive target for multimodal imaging is represented by integrins, that are adhesion protein overexpressed in a variety of neoplasia in course of neoangiogenesis [39–41]. Very recently, Capozza and colleagues [24] have combined ICG with the *α*v*β*3-binding RGD peptide (ICG-RGD) to evaluate in vivo xenograft mouse models of glioblastoma and epidermoid carcinoma, with high and low levels of *α*v*β*3 expression, respectively. ICG-RGD showed improved biodistribution and imaging performances, and a selective integrin-related retention in U-87 MG, but not in A431 tumor cells (Figure 5).

Similarly, EGFR is a relevant biomarker of aggressiveness and resistance to therapy, highly expressed in many types of cancer, and therefore, it appears as a promising target for in vivo imaging. Zhou et al. [42] have investigated the use of an EGFR-specific peptide bonded to Cy5.5, a cyanine dye with spectral features similar to ICG and favorable pharmacokinetic and labeling properties, to detect HCC in a preclinical model by multimodal US-MR-PA imaging. PAI added relevant in vivo molecular information to the US and MRI characterization of tumor growth, as confirmed by immunofluorescence and immunohistochemistry. Targeting strategy could enhance the potential for precision medicine not only through improvement in diagnostic accuracy, but also in therapeutic efficacy. Furthermore, even if the photothermal effect has been the main mechanism exploited in ICG-NIR mediated cancer therapy, more recently antitumoral activity of NPs loaded with ICG has been boosted with synergic drug delivery strategy. For example, Gurka et al. [43] developed high biosafety NPs based on mesoporous silica (MSNPs), loaded with ICG and gemcitabine, improving their specificity for pancreatic cancer by the addition of both chitosan (COS) targeting acidic tumor microenvironment and urokinase plasminogen activator (UPA) that binds to the UPA receptor (UPAR) on tumor cells. In vivo PAI of MSNPs biodistribution showed their specific accumulation into highly metastatic and chemoresistant human pancreatic cancers of orthotopic mouse models, due to the targeting of overexpressed UPAR, as confirmed by ex vivo analysis. Moreover, in vitro studies revealed that coating MSNPs with COS enables the preferential drug release in an acidic environment. This feature could potentially allow to precisely deliver the loaded chemotherapeutic agent into pancreatic tumors, based on the difference in pH between normal and tumoral pancreatic tissues. The authors hypothesized future developments for the MSNPs system such as preclinical assessment of tumor response to the gemcitabine or other chemotherapeutics, and their potential use for improving clinical staging of pancreatic cancer combined with simultaneous treatment. More recently, Liu and colleagues [44] employed a similar targeting strategy in xenograft mouse models of breast cancer for theranostic purposes. They developed folate-receptor-targeted (FA) laser-activatable poly(lactide-co-glycolic acid) (PLGA) NPs loaded with ICG and paclitaxel (Ptx) in order to combine effectively PTT and drug delivery. The folate receptor (FR) is overexpressed on the cell surface of breast, lung, prostate, ovarian, brain, and colorectal cancer, and therefore, it has emerged as the promising molecular target for cancer therapy. Like ICG, PLGA has been approved by the FDA, and in the last years, it has been extensively used for pharmaceuticals synthesis. The PEG-functionalization of PLGA allows to avoid serum protein binding and to enhance retention in targeted cells. These NPs were successfully applied in vivo as contrast agent for dual US-PAI in mice-bearing breast cancer. Furthermore, FA-PLGA-ICG-Ptx NPs showed the capability to selectively damage cancer cells both by the photothermal effect and by Ptx release after laser irradiation, significantly reducing the growth of tumors with high FR expression [44].

### 5.2. Metal-Based Contrast Agents

A lot of nanotechnologies based on golden, silver, ferromagnetic, and other metallic NPs have been developed in order to improve cancer diagnosis and therapy. Taking advantage of strong absorption in the NIR region, related to their peculiarity called the SPR effect (charges on the surface of noble metal NPs due to oscillations according to the electromagnetic field), these nanoplatforms have been successfully implemented as PA contrast agents. Moreover, these NPs have been functionalized allowing to combine a more precise diagnosis with an effective PTT and CHT [132]. Gold NPs (AuNPs) have gained attention as promising PA contrast, due to their greater optical absorption compared to organic agents. AuNPs are characterized by various shapes, like sphere, rods, shell, or prism, and different sizes, which in turn confer them a wide range of absorption wavelengths (650–1100 nm) in a window with high contrast compared to biological tissues [45]. Furthermore, AuNPs can be easily modified with different surface moieties to increase targeting efficiency, enhancing the imaging contrast and PAI-based therapy. In this view, AuNPs conjugated with gadolinium (Gd^3+^) have proven to be a useful multimodal contrast agent in the A549 tumor bearing mice. Indeed, this nanoplatform showed a significant signal amplification for CT imaging and reasonable r1 relaxivity for MRI, establishing a potential tool for translational cancer diagnosis [46]. Further improvements of AuNPs applications have been achieved through advances of their simultaneous diagnostic and therapeutic capability. Using the ligand-receptor pathway, the conjugation with FA enabled the internalization of AuNPs in HeLa cancer cells, allowing to visualize a high accumulation in tumor-bearing mice [47]. In addition, following laser irradiation triggered strong shock waves by FA-AuNPs, resulting in cancer cell ablation. Recently, PA-guided PTT has been synergically combined to gene therapy, exploiting siRNA mediated gene-silencing effect on cancer cells. AuNPs, coupled with Zn(II)-dipicolylamine (Zn-DPA) were able to specifically bind siRNA, leading to an alternative strategy for efficient and safe siRNA delivery into targeted cancer cells. Through the use of theranostic nanocomplexes, with both PAI and PTT activities, it may be possible to overcome siRNA issues related to their poor pharmacokinetic, cytotoxicity, and low cellular internalization following systemic administration. Moreover, the combination of siRNA agents and PTT could potentially provide enhanced antitumor efficacy compared to conventional single treatments. In vivo PAI using Zn-DPA-AuNPs loaded with antipolo-like kinase 1 silencing siRNA (siPLK1) showed favorable biodistribution and maximum signal in the prostate carcinoma xenograft mouse model at 24 hours after injection. Thereafter, to investigate their antitumoral efficacy, PTT with 808 nm laser irradiation was performed using both free Zn-DPA-AuNPs and the siPLK1-Zn-DPA-AuNPs. The combination of both siRNA gene silencing and PTT was effective to produce tumor regression, probably due to induced tumor cells apoptosis, compared to growth delay obtained by single therapy [48].

An alternative method to functionalize AuNPs for cancer theranostic is to create a pH-sensitive nanoplatform, exploiting the acidic tumor microenvironment in contrast to healthy tissues. To this aim, the biocompatible, biodegradable, and pH-responsive copolymer PAsp(DIP)-b-PAsp(MEA) was synthesized and self-assembled into PEGylated micelles, loaded with golden nanocages (GNCs) and doxorubicin (DOX) (D-PGNC) [49]. In vivo studies have been performed by D-PGNC IV injection in a xenograft ovarian cancer mouse model, in order to evaluate their potential for PAI and to develop a chemotherapeutic system triggered by both NIR irradiation and the tumor pH. A strong PA signal in the tumor was displayed at 8 hours after D-PGNC IV injection compared to control mice receiving PBS. Moreover, although in this experiment PGNC with laser irradiation and D-PGNC alone showed an evident therapeutic outcome on tumor growth, the DOX-loaded pH-sensitive micelles combined with PTT achieved tumor ablation, presumably promoting DOX penetration into cancer cells without systemic side effects [49].

Another pH-sensitive drug delivery system was developed using gold nanostars (GNSs) loaded with both ICG and calcium carbonate (CaCO_3_) (GNSs-CaCO_3_-ICG) [50]. The CaCO_3_ is able to self-dissociate in acidic microenvironment, allowing the preferential release of ICG in specific tumor types, resulting in a more specific diagnostic PA signal. Moreover, the GNSs photothermal capability and the ICG photodynamic properties can be conveniently exploited for a boosted photodynamic (PDT) and PTT approach. In vivo targeting specificity and antitumor efficacy of GNSs-CaCO_3_-ICG were tested in MGC 803 gastric carcinoma bearing mice. Combination of ICG with GNSs-CaCO_3_ promoted its selective accumulation in the tumor region, with a peak enhancement at 24 hours after IV injection, suggesting their potential use as the induced drug delivery system. In addition, dual PTT/PDT using GNSs-CaCO_3_/ICG exhibited a strong synergic effect in inhibiting tumor growth, compared to the animal group injected with GNS or ICG alone [50].

Similarly, smart AuNPs (SAuNPs) have been designed to form aggregate in mildly acidic microenvironment, resulting in redshift of the NIR absorption spectrum based on the SPR effect. SAuNPs can selectively accumulate in specific tumor sites without need of further structural modifications, and therefore, they could be an interesting tool for both tumor monitoring and treatment by multimodal PAI-PTT [51]. Furthermore, SAuNPs could be potentially incorporated in microbubbles (Mbs-SAuNPs) in order to favor their local release in tumor sites by US sonoporation. Such theranostic platform has been tested in U-87 MG bearing mice, highlighting its usefulness for tumor detection by CEUS and achieving successful cancer cell ablation after laser irradiation [52].

Dual mode US-PAI has been used also by Li and coworkers, who developed a nanoplatform composed by AuNPs and liquid perfluorohexane (PFH) that provides a microbubble contrast medium. Moreover, Au-PFH-NPs have been conjugated to a monoclonal antibody against melanoma-associated antigen (MAGE), to specifically target melanoma cells [53]. Preliminary results in B16 melanoma tumor-bearing mice showed that after IV MAGE-Au-PFH-NPs injection, an early PA peak signal appeared in the tumor site at 2 hours and persisted until 24 hours, highlighting a favorable kinetic for cancer imaging. After NIR irradiation, the AuNPs-mediated temperature increase triggered the PFH phase change from liquid to gaseous state, allowing CEUS imaging to improve tumor detection [53]. Further studies are needed to implement the use of MAGE-Au-PFH-NPs as a promising approach for noninvasive melanoma targeting and treatment.

A different method inducing self-aggregation of AuNPs in tumor lesions is also represented by binding with thermosensitive peptides to promote tumor targeting [54]. Sun and colleagues developed a biocompatible and thermally sensitive elastin-like polypeptide (ELP) and applied AuNPs conjugated with this agent for in vivo multimodal CT-PAI.

The authors hypothesized that ELP-AuNPs could form aggregates into neoplasia as the intratumoral temperature is assumed to be higher than ELP temperature cutoff (23°C) for phase transition. After intratumoral injection in a C8161 melanoma xenograft mouse model, the ELP-AuNPs PA signal resulted homogenous and increased over time, unlike control mice injected with PEG-AuNPs or PBS, and in agreement with CT enhancement. Furthermore, upon laser irradiation, tumor lesions in the mice group injected with ELP-AuNPs were eradicated without recurrence. The authors suggested that ELP-AuNPs thermosensitivity could represent an advantage over other tumor targeting strategies since it is independent from the EPR effect and tumor microenvironment [54].

Improvement of therapeutic efficacy has been reached using a multifunctional system including AuNPs associated with mesenchymal stem cells (MSC), exploiting MSC migration into tumor site for targeted drug release and PTT. Stem cells tumor cell tropism, mainly exerted through stromal cell-derived factor-1- (SDF-1-) CXCR4 receptor interaction, have been used in combination with AuNPs, for theranostic purposes [55].

To this aim, Xu et al. proposed a novel nanoplatform based on MSC loaded with plasmonic-magnetic lipid NPs, DOX, AuNPs, and iron oxide (IO) NPs (MSCs-LDGI).

In vitro analysis demonstrated that LDGI are internalized by MSCs with low cytotoxicity and that IONPs were able to upregulate the CXCR4 expression on the MSCs, improving tumor specificity. Based on encouraging results of cellular studies, the in vivo MSCs-LDGI antitumor effect was tested in a xenograft mouse model of triple negative breast cancer, with high metastasis and recurrence rate.

In vivo PAI after intratumoral injection revealed the ability of MSCs-LDGI to migrate into the tumor area compared to bare LDGI. Overall, MDA-MB-231 tumor-bearing mice treated with of MSCs-LDGI and subsequent laser irradiation exhibited the greatest antitumor efficiency compared to mice receiving single treatment, achieving tumor eradication without recurrence during the following 2 weeks. Similarly, after MSCs-LDGI IV administration, PA images showed that mesenchymal stem cells are crucial for an optimal distribution of AuNPs in tumor site and to obtain the best therapeutic results [55].

Despite their potential usefulness in PAI and PTT, AuNPs have some limits like cost and chemical instability due to their complex nature, also affecting in vivo pharmacokinetics.

**Figure 6:**
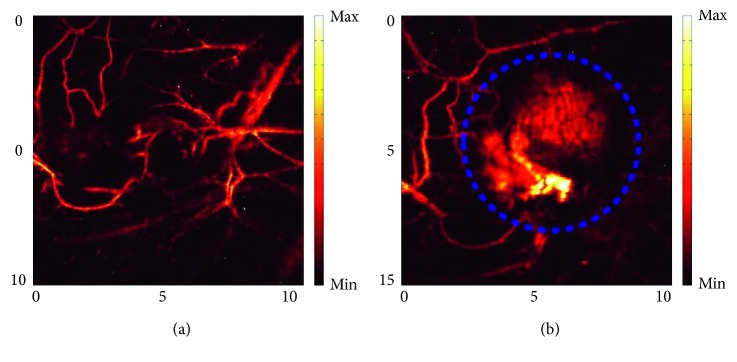
In vivo PAI of MDA-MB231 xenograft mouse model before and 1 hour after IV injection of Pd-COS-RGD (adapted with permission from Bharathiraja et al. [56], CC BY-NC-ND license, http://creativecommons.org/licenses/by-nc-nd/4.0/). (a) Before injection; (b) after injection.

Another noble metal recently proposed as theranostic agent alternative to AuNPs to overcome these issues is palladium (Pd), thanks to its lower costs and stable plasmonic properties after repeated laser exposures. Only few studies have investigated its potential in vivo applications. For example, Pd combined with COS and RGD has been currently applied for theranostic purposes in MDA-MB-231 xenograft mouse model [56]. After IV administration, Pd-COS-RGD accumulated in the tumor region, leading to a significant PA signal compared to control mice. Interestingly, the authors observed that photothermal efficiency of Pd was comparable to those of AuNPs, but with higher stability. In vivo PTT using Pd-COS-RGD induced an increase of tumor temperature to 50°C over 2 minutes, leading to a complete tumor ablation [56] (Figure 6).

Further studies have been based on the research of other cost-effective metal-based nanomaterials with high theranostic performances and facile synthesis, such as copper-based semiconductors, bismuth-based NPs, transition metal-based nanomaterials, and magnetic IONPs [65]. Among these, copper sulfide (CuS) NPs have acquired increasing interest in cancer diagnosis and therapy, due to their advantageous properties, including good NIR optical absorption, its molar extinction coefficient, efficient photothermal conversion, and good biodistribution, in addition to relative economicity and low toxicity [57, 58]. Moreover, CuSNPs are suitable to be functionalized for dual-modality imaging to improve theranostic specificity and sensitivity. In this regard, Gd^3+^ ions chelated diethylenetriaminepentaacetic acid (DTPA-Gd^3+^) have been conjugated with bovine serum albumin (BSA) and CuSNPs, to act as the contrast agent for tumor detection by PAI and MRI [57]. In vivo T1 weighted MRI in glioblastoma-bearing mice showed increasing signal during the 24 hours following the DTPA-Gd^3+^-BSA-CuSNPs IV injection, in agreement with the PA enhancement, allowing a clear identification of tumor edges. Taking advantages from high sensitivity and deep tissue penetration of PAI associated with high resolution of MRI, this dual nanoprobe could represent a useful tool for early detection of cancer and guidance therapy [57].

In spite of the PAI capability for deep tissues analysis, a limitation of imaging in oncology may be represented by the background signal produced by endogenous absorbers, which in turn could lead to reduced signal-to-noise ratio (SNR). In particular, hepatic neoplasias appear difficult to diagnose since the liver is a highly vascularized organ, and it is involved in nonspecific accumulation of several imaging probes. Therefore, it could be advantageous to introduce contrast media with low background signal and deep penetration into tissues. To overcome the drawbacks, CuSNPs with excitation wavelengths in the range of 1000–1700 nm (NIR II), conjugated with BSA and RGD, have been tested for the first time in a orthotopic mouse model of HCC. In vivo PAI showed high SNR and liver peak enhancement at 24 hours after CuS-BSA-RGD NPs IV injection, allowing clear tumors detection, in contrast to CuS-BSA-NPs, and with negligible toxicity. These results could be ascribed to the high PA signal from CuS-BSA-RGD NPs and to the low light hepatic absorption of the liver upon 1064 nm wavelength, as well as to the excellent selectivity of the RGD peptide for integrin receptors, overexpressed in tumor vessels. Collectively, this study highlighted the potential utility of the NIR II contrast agent to investigate in vivo cancer disease in deep organs, due to the low optical absorption of the biological tissues at these specific wavelengths [59]. Advances in functionalization procedures may help to improve not only tumor detection but also therapeutic efficacy. One of the major challenges for cancer therapy is to prevent recurrence from residual cancer cells after surgery or therapy. Li et al. exploit photothermal conversion feature and biocompatibility of CuSNPs, in order to develop a nuclear-targeted nanoplatform inducing cancer cell ablation. CuS enclosed into MSNPs has been modified with RGD to specifically recognize angiogenic tumor vessels and with the TAT peptide for specific nuclear targeting [58]. After local and IV CuS-MSN-TAT-RGD injection in HeLa tumor-bearing mice, a 5-minute PTT was performed after 8 hours at 980 nm wavelength, resulting in tumor eradication, without recurrence in a 14-day monitoring period. Assuming the significant increase of nuclear temperature exerted by the irradiated CuSNPs, together with the selectivity of tumor and nucleus targeting by the RGD and the TAT, respectively, this nanoplatform could constitute a promising tool for cancer therapy avoiding recurrences [58].

Other novel copper- and sulfide-based theranostic nanoagents, like Cu-Ag_2_S and iridium (IrS_*x*_), have been proposed, but the low photothermal conversion efficacy has limited their use. In a recent study, the combination of biocompatible Cu_2_-*x*S and Ag_2_S into a multifunctional nanoplatform for PAI-PTT has been described to overcome the limitations of each single agent. Polyvinylpyrrolidone- (PVP-) stabilized Cu-Ag_2_S NPs were injected IV in a 4T1 tumor xenograft mouse model, showing a maximum PA signal after 6 hours after injection upon 808 nm laser irradiation. As expected on the basis of in vitro analysis, the Cu-Ag_2_S-PVP after laser irradiation provided an effective inhibition of tumor growth. The relevant efficiency of tumor eradication, in conjunction with their biocompatibility, makes Cu-Ag_2_S-PVP NPs interesting for advanced studies in cancer therapy [60].

As a sulfide metal, Irs is able to provide excellent X-ray attenuation and, at the same time, an efficient photothermal conversion in the NIR region. Recently, IrS_*x*_-PEG-FA NPs were IV injected in the xenograft HeLa tumor mouse model [61], resulting in a strong PA signal in tumor site after 24 hours, in agreement with CT enhancement, showing a good correlation between Hounsfield units and IrS_*x*_ concentration. Furthermore, IrS_*x*_-PEG-FA NPs, conjugated with anticancer drug camptothecin, exhibited both photothermal effect and pH-photothermal-responsive drug release properties. In vivo combined PTT-CHT induced almost a complete tumor eradication, more efficiently than the single therapy administered in the control group. In summary, IrS_*x*_-based NPs would be further investigated to develop future applications in integrated nanoplatform for tumor multimodal diagnosis and ablation [61].

Similarly, among semimetal elements, bismuth (Bi) is characterized by a high X-ray attenuation coefficient in association with high photothermal efficiency, due to plasmonic properties and biocompatibility. Therefore, Bi has attracted more interest in multimodal cancer imaging and therapy. In addition, Bi presents strong absorption in the NIR II, with the advantages of improved SNR and deep penetration into tissues.

In recent years, ultrasmall Bi NPs, labeled with a peptide LyP-1, have showed high tumor uptake, offering a potential theranostic contrast agent for PAI, CT, and combined PTT-RT [62]. To corroborate this, Bi-LyP-1 NPs were IV injected in a 4T1 breast cancer-bearing mice, showing significant PA signal intensity at 8 hours after their administration. Further studies using RT improvement and dual-modal imaging of Bi-LyP-1 NPs demonstrated the most relevant results for tumor growth inhibition in Bi-LyP-1 NPs treated mice with both exposures to 1064 nm wavelength and 4 Gy irradiation. In this regards, thanks to the ability to absorb both X-ray and NIR-II laser, the presented nanoplatform has gained interest in theranostics [62]. A similar strategy in the same breast cancer model was conducted by Yang and colleagues [63] which have drawn up the PEG-modified polypyrrole- (PPy-) coated Bi nanohybrids (Bi-PPy-PEG NPs) for multimodal CT-PAI and PTT with high biosafety. Both PAI and CT performances have been explored after Bi-PPy-PEG NPs intratumoral injection, showing PA signal enhancement proportionally to the NPs injected concentration, and strong CT values with higher contrast than the iohexol conventionally used in clinic. In order to evaluate PTT efficacy, 24 hours after Bi-PPy-PEG NPs IV injection, 4T1 tumor-bearing mice were irradiated by 808 nm NIR laser, resulting in tumor growth inhibition without evident toxicity signs. Importantly, Bi-PPy-PEG NPs offer improved photothermal conversion upon short and repeated NIR irradiation cycles and as the CT contrast agent showed superior imaging features than currently used iodine-based agents [63]. Following the promising results in multimodal imaging and therapy obtained with Bi-based NPs, very recently Wu and coworkers [64] have explored the efficacy of Gd-PEG-Bi NPs as novel nanoplatform for MRI-CT-PAI and PTT in a glioma mouse model after their IV injection. In vivo MRI evidenced a peak enhancement at 3 hours after administration, highlighting a much longer retention time in tumor than the gadopentetate dimeglumine routinely used in the clinic. Moreover, the potential use of these NPs as the CT contrast agent has been confirmed in vivo, revealing significantly enhanced CT signal in tumor lesion 1-hour after injection. Finally, as for MRI, PA signal showed a peak enhancement at 3 hours after injection. Furthermore, the authors demonstrated that upon 808 nm laser irradiation, tumor growth was effectively inhibited by PTT, and xenografts were completely ablated. Overall, authors suggested that Gd-PEG-Bi NPs may potentially constitute an integrated approach as the therapeutic agent whose efficacy can be conveniently monitored through trimodal imaging [64]. Among transition metals, titanium (Ti) has been already widely used in tissue engineering due to its excellent biocompatibility. Taking advantage of its strong NIR absorption, Qian et al. [66] investigated for the first time titanium disulfide (TiS_2_) conjugated with PEG as a new photothermal agent for cancer therapy [66]. PAI in 4T1 tumor-bearing mice, using IV TiS_2_-PEG NPs injection and 808 nm NIR laser irradiation, resulted in evident signal enhancement at 24 hours, highlighting their distinct accumulation in lesion due to the EPR effect. Likewise, in vivo PTT exposing xenografts to the same NIR wavelength produced early tumor ablation 1 day after treatment, without recurrence after 10 days. This study paved the way for the development of other Ti-based compounds for theranostic applications in cancer research [66]. More recently, PAI-PTT performances of Ti-nitride NPs (TiN NPs) were confirmed in HeLa tumor-bearing mice following their IV injection. An increased PA signal over time was observed, peaking at 24 hours after administration. Thereafter, PTT exerted an evident anticancer response within 15 days of treatment, leading to complete tumor eradication without systemic side effects [65].

In addition, Ti-based oxide nanomaterials (TiO_2_) have showed the potential for PDT after UV light or radio luminescence exposure, with the advantage of higher biocompatibility over heavy metal-based compounds previously described. Nevertheless, some issues, related to limited penetration depth of UV light in biological tissues and to poor cost-yield ratio of radio luminescence, has encouraged to develop TiO_2_ NPs with high NIR absorption like niobium-doped TiO_2_ NPs (Nb-PEG-TiO_2_) [67]. In particular, photothermal properties can be dynamically modulated by changing the molecular Nb doping levels, while PEGylation confers high stability and biocompatibility to the nanosystem. 30 minutes after intratumoral injection in HeLa tumor-bearing mice, Nb-PEG-TiO_2_ generated an evident PA signal. Moreover, subsequent exposure to 1064 nm laser for 10 minutes was able to prove Nb-PEG-TiO_2_ efficiency for PTT, resulting in tumor ablation [67]. Similar to Ti, magnetic IONPs have been already widely used in biomedical research, especially in the field of MRI, and in last decade have been optimized also for PAI and PTT in mouse models of cancer disease. Overall, several studies using magnetic IONPs for theranostic purposes have focused on the synthesis of effective and biocompatible materials at the same time for multimodal imaging, developing new hybrid materials composed by both metal ions and organic polymers. For example, Monaco et al. [68] developed Fe_3_O_4_ NPs composed by a silica layer and a gold shell conjugated to FA for cancer cells targeting. In addition, Fe_3_O_4_-SiO_2_-Au NPs have been coated with an organic ligand, in order to be included into biocompatible polymeric micelles, allowing to build targetable nanostructures for dual-modality imaging. MRI images, obtained following Fe_3_O_4_-SiO_2_-Au PMs-FA NPs IV injection in a xenograft mouse model of ovarian cancer overexpressing FR, showed a relevant *T*_2_ weighted contrast enhancement in tumor at 4 hours after injection. In agreement, the PA peak signal was found at the same MRI time point, likely related to the FR targeting in the lesion [68].

For advanced purposes, IONPs applications for imaging guided tumor treatment by PTT and synergistic CHT have been investigated. Jin et al. [69] developed coordination NPs (CPN) mixing a solution of FeCl_3_ and gallic acid modified with PEG and ^64^Cu isotope for multimodal imaging and cancer therapy. In vivo PET imaging performed in 4T1 tumor-bearing mice after ^64^Cu-Fe-GA-PEG CPNs IV administration showed a higher uptake of NPs compared to the control group injected with ^64^Cu-Fe-GA CPNs, demonstrating that PEGylation promotes their tumoral tropism via EPR. In parallel, the efficacy of such CPNs for PAI with 808 nm NIR laser and *T*_2_ weighted MRI was also demonstrated, highlighting a clear contrast enhancement at the tumor site. Finally, the best therapeutic result was obtained in tumor-bearing mice treated with Fe-GA-PEG CPNs and exposed at 808 nm NIR laser for 5 minutes, achieving the complete eradication of cancer lesions [69]. Another hybrid NPs taking advantages of excellent biocompatibility and high photothermal conversion efficacy of eumelanin (euMel) in association with IONPs was developed for in vivo MRI-PAI and therapeutic purposes [70]. PAI and MRI were performed in U-87 tumor-bearing mice after interstitial injection of euMel-Fe_3_O_4_ NPs, obtaining a significant signal enhancement of the tumor site for both imaging methods. In addition, in vivo cancer therapy efficacy was proved exposing tumor-bearing mice to 808 nm laser irradiation for 5 minutes. Combining euMel-Fe_3_O_4_ NPs with laser irradiation, a 51.1°C final temperature of tumors was reached, followed by their eradication without surrounding tissue damage. These hybrid NPs have combined the favorable paramagnetic properties of IONPs with the contrast efficacy and biodegradability of an endogenous chromophore, allowing a highly sensitive multimodal imaging without evident toxic effects [70]. Superparamagnetic IONPs (SPIONPs) are characterized by improved biocompatibility and biosafety, which together with efficient photothermal conversion make them a promising tool for in vivo PAI and PTT.

To shed more light on SPIO therapeutic capability, a NIR light-controllable, targeted, and biocompatible drug delivery nanoplatform have been proposed.

PFH-PTX-PLGA-SPIO NPs targeting human epidermal growth factor receptor 2 (HER-2) have been tested as the theranostic agent in a xenograft mouse model of SKBR3 breast cancer [71]. In this nanoplatform, PLGA incorporates SPIONPs, the chemotherapeutic, and it is conjugated with both liquid PFH and the Herceptin HER-2 ligand, allowing for selective accumulation in the tumor site. Furthermore, upon 808 nm NIR laser irradiation, SPIO converts NIR light in thermal energy, resulting in cancer cell ablation. Moreover, overheating triggers the optical droplet vaporization of PFH, that exerts a double function: generating gas microbubbles for CEUS and contributes to the PTX release. This theranostic strategy allowed to monitor tumor progression with high accuracy by US-PAI, reaching at the same time a complete lesions eradication with improved specificity than conventional chemotherapeutic protocols and without evident side effects. Moreover, the PLGA, SPIO, and Herceptin components have been approved by the FDA, encouraging their clinical translation [71].

### 5.3. Carbon-Based Contrast Agents

In the last decade, carbon (C)-based NPs have gained growing attention in several field of biomedical research, including cancer diagnosis and therapy, thanks to their marked photoabsorption, photostability, solubility, and biological compatibility.

In particular, C nanotubes (CNTs) are able to be internalized from cells without any functional group on their surface; nevertheless, they provide multiple sites for covalent or noncovalent attachment of different targeting moieties. Moreover, functionalized CNTs may be more efficiently employed to deliver a variety of molecules like peptides, antigens, nucleic acids, and drugs inside cancer cells for theranostic purposes.

Based on their physical features, CNTs are mainly classified in single-walled CNTs (SWCNTs) consisting of a cylinder with a unique sheet and diameter range of 0.2–2 nm, and multiwalled CNTs (MWCNTs) which are characterized by several layers and a diameter range of 2–10 nm [72, 73]. The specific advantages of SWCNTs, including optical absorption of all the visible light spectrum, high thermal conversion, and maximum size of 2 nm, make them more attractive for PAI. Instead, due to their larger size, MWCNTs are more suitable for delivery of large biomolecules such as DNA plasmids. Furthermore, their PAI signal can be enhanced through combination with other contrast agents like AuNPs or ICG [72, 73].

In this perspective, several investigations have focused on CNTs conjugation with optical dyes like ICG, improving at the same time the PA signal and biosafety of contrast molecules. Among first, Koo et al. [74] has enhanced PA sensitivity of SWCNTs by ICG attaching. In vivo PAI in healthy rats has demonstrated the ICG-SWCNTs capability to map SLNs and to visualize urinary bladder based on renal excretion. Moreover, ICG-SWCNTs reached a 4 time greater PA signal intensity than plain SWNTs. Therefore, the authors suggested their potential utility to identify SLNs in breast cancer patients and to perform cystography for cancer diagnosis.

Similarly, Zanganeh et al. [75] have tested the feasibility to assess tumor margins and size in a xenograft mouse model of 4T1 breast cancer by ICG-SWCNTs, highlighting their potential application to guide surgical resection of neoplasia. In addition, de la Zerda and colleagues [76] have functionalized ICG-SWCNTs with cyclic RGD peptides, allowing *α*v*β*3 integrins targeting of U-87 MG tumor xenografts in living mice, focusing on improved imaging performances compared to simple SWCNTs-RGD.

Like metallic-based NPs, biodegradability is a relevant concern for CNTs clinical translation, and different synthesis strategies has been proposed to overcome this issue. For example, Lee et al. [77] demonstrated by in vitro UV-Vis absorbance test that the degradation of nitrogen-doped carbon nanodots (N-CNDs) was improved. In vivo experiments in a rat model revealed that N-CNDs allowed to detect SLNs 30 minutes from intradermal injection, aiding for metastatic cancer diagnosis with low toxicity, and thereafter, they are eliminated by renal clearance.

Another common issue of NPs is represented by their rapid clearance after IV administration. To circumvent this limit, Xie et al. [78] developed SWCNTs characterized by long circulation time and capability of both FL-PA imaging and photothermal ablation of tumors. The SWCNTs were coated with Evans blue (EB), improving both their water solubility and binding with serum albumin, which in turn prolong their circulation. Moreover, this SWCNTs-based system was conjugated with an albumin-photosensitizer chlorin e6 (Ce6) complex, enabling dual-modality imaging of tumors and improving tumor treatment. This multifunctional SWCNTs allowed to detect tumor sites in squamous cells carcinoma-bearing mice, showing a fluorescent PA signal peak at 24 hours after IV administration. Moreover, albumin-Ce6-SWCNTs have demonstrated tumor ablation efficacy after irradiation, using a combined PTT-PDT approach. The authors concluded that combining FL and PA imaging could provide complementary information for tumor monitoring and can optimize therapeutic planning.

In the last years, theranostic applications of CNDs are emerging due to their high biosafety, photostability, and targeting properties. Several novel synthesis CNDs, coupled with other elements such as sulphur, phosphorus, and N to modify their spectral emission, have showed high thermal efficiency and have been investigated in mouse models of human cancer for both FL-PA imaging and PTT [79]. After IV administration, these innovative CNPs have showed desirable biodistribution in tumoral tissues through EPR and renal clearance, making them promising for future clinical translation [79].

In agreement, Parvin and Mandal [80] have demonstrated the utility of dual wavelengths emitting CNDs codoped with phosphorus and N (PN-CNDs) for FL-PA imaging of a xenograft mouse model of human gastric carcinoma. After IV injection, the PN-CNDs revealed a significant uptake in the tumor site at different time points, showing a rapid accumulation within 12 minutes, and a progressive rise of the FL-PA signal, peaking at 3 and 6 hours after injection for green and red emitted light, respectively.

Nowadays, the combination of PDT and PTT has been proved to give a better anticancer response compared to single strategy, allowing to use lower radiation dose. Moreover, this approach avoids toxic side effects and drug resistance compared to conventional CHT and RT.

Very recently, Yang and coworkers [81] investigated the potential of CNPs doped with zeolitic imidazolate framework-8 (ZIF-8) as photothermal and photosensitizer, exploiting the simultaneous production of heat and ROS after their NIR irradiation. Interestingly, ZIF-8-CNPs synthesis has proven to be relatively simple, economic, and efficient. Furthermore, in vivo experiments highlighted good PAI contrast in a mouse model of human lung carcinoma and their suitability for oncotherapy, positively related to the increase in NPs size. In light of their overall performances, the authors hypothesized that ZIF-8-CNPs are expected to have wide clinical applications in a near future.

Analogous attractive results have been obtained by Xu et al. [82] in a xenograft mouse model of 4T1 breast cancer using newly developed supra-CNDs. This contrast agent is based on an emerging technique to engineer CNDs through electrostatic interactions and hydrogen bonding, to obtain a strong absorption band from visible light to NIR and efficient photothermal conversion.

Using the same mouse model, Jia and colleagues have described an innovative bimodal FL-PA imaging approach to guide synergistic PDT-PTT of cancer [83], employing the *Hypocrella bambusae* (HB) biomaterial as the precursor for the preparation of CNDs-based phototheranostic agents. HB-CNDs offer several advantages including a relatively simple and cheap synthetic procedure, adequate photostability, good water solubility, broad absorption, red-light emission spectra, and excellent biocompatibility. In vivo experiments have demonstrated the feasibility of FL-PA imaging by HB-CNDs and encouraging results through bifunctional treatment, with optimal time point for both imaging and phototherapy at 8 hours after HB-CNDs IV injection.

The same research group proposed another type of multifunctional CNDs coupled with gold nanorods (GNRs) and a silica scaffold (SiO_2_). Globally, in GNRs-SiO_2_-CNDs, the GNRs exert both PAI contrast and photothermal effect, while the CNDs have been exploited for FL imaging and PDT. SiO_2_ aids improvements in both chemical and optical stability of GNRs and CNDs. GNRs-SiO_2_-CNDs have been tested in a mouse model of skin melanoma, showing a favorable in vivo biodistribution, high contrast, and spatial resolution to visualize the tumor for subsequent thermal ablation. Overall, these pilot studies provided promising perspectives of effective and safe application of these phototheranostic agents in biomedicine.

Other light-absorbing molecules have been combined with CNDs to develop new nanomaterials, providing multimodal imaging together with therapeutic strategies. For example, Wu and coworker [84] have recently prepared CNDs bonded with a porphyrin macrocycle (PCNDs), considering its desirable properties such as high radiation absorption in the UV, visible, and NIR spectra, as well as its favorable photothermal efficiency. These biocompatible NPs nanomaterials are characterized by easy synthesis and could be helpful for PAI with enhanced performances compared with conventional optical imaging modalities. Furthermore, PCNDs have been functionalized with cetuximab (C225-PCNDs) for targeting cancer cells overexpressing EGFR. Moreover, intratumoral injection of C225-PCNDs in a mouse model of human breast cancer has demonstrated an improved capability for both tumor detection and ablation. Such in vivo results indicate that C225-PCNDs could represent a promising platform for cancer theranostics. Finally, in the past decades, all carbon nanomaterials, including graphdiyne (GDY), have gained growing interest for exploring their biomedical applications as potential photothermal agents, in light of their excellent ability of energy conversion. Li et al. [85] described the first in vivo use of GDY-based PEGylated nanosheets (GDY-PEG) for simultaneous PAI and PTT in a xenograft mouse model of 4T1 breast cancer. After intratumoral injection of GDY-PEG and laser irradiation, strong PA signal and efficient photothermal ablation were observed. Moreover, the biodistribution of GDYs-PEG has been studied, revealing a peak of tumor enhancement at 12 hours after IV administration, positively correlated with the GDYs-PEG concentration. On the basis of these findings, the authors hypothesized that GDYs-PEG could provide new insights for the development of novel theranostic tools.

Nevertheless, in vivo applications of all carbon NPs are still in infancy. Very recently, other all carbon NPs [133] have been synthetized, like N-doped graphene (N-G) CNDs, with the advantages of low cost, low cytotoxicity, and higher photothermal conversion efficiency than other C-based nanomaterials currently available. In addition, N-G-CNDs conjugated with FA have enabled for active targeting of several tumor cell types in vitro, aiming future preclinical employ for in vivo dual-mode FL-PA imaging and PTT.

### 5.4. Semiconducting Polymer Nanoparticles

Recent advances have been made for cancer PAI and PTT using semiconducting polymer (SP) NPs, an emerging group of contrast agents characterized by optical features, biocompatibility, photostability, and functionalization potentially more advantageous for in vivo applications compared to the most widely used organic dyes, metallic NPs, and carbon nanomaterials [86].

The remarkable optical features of SPNPs are mainly related to the presence of delocalized electrons, while PA signal enhancement has been achieved by modifying their polymeric structure and designing activatable molecular probes [87].

Initial investigations on the use of SPNPs for in vivo PAI were conducted by Pu and colleagues [88] that highlighted the advantages over SWNTs or GNRs, based on a wider and more stable PA signal, and higher detection sensibility. In a following study, the same author and coworkers [89] highlighted the feasibility of diketopyrrolopyrrole- (DPP-) based SPNs for in vivo PAI on a HeLa xenograft mouse model. After IV administration, DPP-SPNs exhibited a significantly higher PA signal in tumor than surrounding tissues, presumably due to extravasation of SPNs through cancer blood vessels. These studies provided the first insights about the potential of SPNs for high-resolution PAI in oncological research.

Further improvements in biodistribution of amphiphilic polymers containing DPP-SPNs have been obtained through PEGylation [90], overcoming also the potential issue of their dissociation. In a proof of concept study, the ability of these amphiphilic SPNs for in vivo tumor imaging was demonstrated at 24 hours after IV injection.

**Figure 7:**
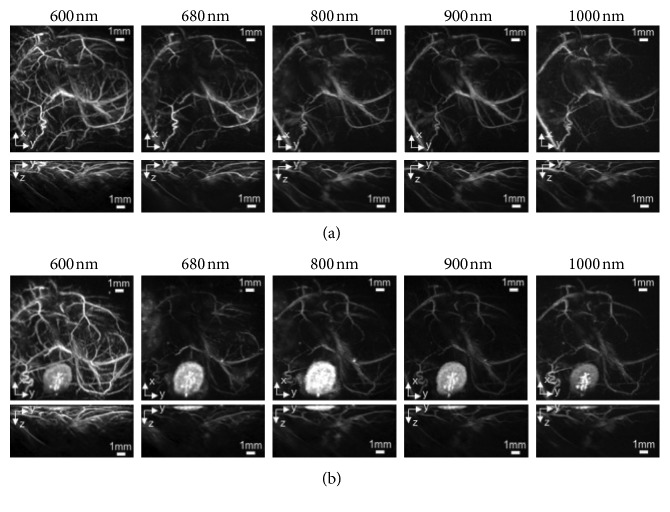
Multiwavelength in vivo PAI (a) before and (b) after subcutaneous injection of indigoid *π*-conjugated SPNs, showing their ability to distinguish PA signal generated by the contrast agent and vasculature for potential oncological applications (adapted with permission from Stahl et al. [92], CC BY-NC-ND license, http://creativecommons.org/licenses/by-nc-nd/4.0/).

Attempts for advances in diagnostic specificity and sensitivity are also in progress through implementation of multispectral PAI. Zhang and colleagues [91] developed semiconducting polymer dots (Pdots), which include in their structure isoindigo (IID) and 4,7-dithien-2-yl-2,1,3-benzothiadiazole (DTBT) as electron acceptors. This chemical modification, consisting in incorporation of an electron-deficient element into the SPNs structure, is known as the “self-quenched process,” and allows to enhance both PA signal intensity and heat generation. SPNIID-DTBT-based Pdots are characterized by strong optical absorption in the 500–700 nm spectral range, opening promising perspectives for better discrimination of tumoral tissue from blood vessels. In vivo US-PAI preliminary results highlighted the possibility to detect a clear PA signal after injection of gelatin containing a Pdots solution into the dorsal subcutaneous tissue of nude mice. Moreover, by applying such gelatin mixture onto mice ears, the authors demonstrated the feasibility to discriminate between PA signal derived by Pdots solution from that by surrounding vessels. In agreement, a new class of indigoid *π*-conjugated SPNs have showed maximum absorption in the optical window where endogenous chromophores have low absorption [92]. In vivo PAI after subcutaneous injection of such SPNs in mice has proven the wavelength-dependent capability to distinguish the PA signal generated by the contrast agent from that by vasculature (Figure 7).

Despite enhanced sensitivity of last-generation PAI agents, the deep tissue penetration capability is a current challenge, depending on the excitation wavelength used, the light scattering in different tissues, and the absorption from endogenous Hb. In this perspective, Fan et al. [93] developed perylenediimide-based (PDI) NPs and demonstrated their efficiency for specific and noninvasive brain imaging in an orthotopic glioblastoma mouse model, with better tumor penetration than silica-coated AuNPs.

Moreover, SPNs are highly prone to synthetic modification for targeting specific receptors, through conjugation with ligands such as antibodies, RGD peptide, FA, and biotin or for drug delivering [86].

In this perspective, self-quenched strategy has been further improved for in vivo application by Xie and coworkers [94] through conjugation with a cyclic-RGD peptide, allowing evaluation of *α*v*β*3 integrin receptor expression in a xenograft mouse model of 4T1 breast cancer.

In addition, efforts for the development of more sensitive PAI agents have been made applying on DPP-SPNs a surface engineering method like coating with a silica layer (SiO_2_), to amplify the FL-PA signal and guarantee efficient photothermal conversion at the same time [89]. DPP-SPNs-SiO_2_, conjugated with PEG and cyclic RGD, have resulted suitable for targeting of xenograft 4T1 breast tumors in living mice after IV administration, showing a high PA signal to background ratio.

Chemical structure of different SPNs influences not only PA brightness but also photothermal conversion efficiency for cancer treatment, and recent preclinical studies have explored their theranostic performances to provide molecular insights into the design of effective SNPs for both PTT and PAI in oncology [95].

Chemophotothermal theranostic nanoplatforms are emerging as a promising tool for cancer therapy improvements by a synergistic approach. To overcome the current issue of cancer resistance to conventional CHT, amphiphilic PEGylated poly(cyclopentadithiophene-alt-benzothiadiazole) (PEG-PCB) SPNs have been recently developed as multifunctional nanocarrier integrating DOX delivery with thermal ablation [96]. After IV injection, PEG-PCB-SPNs were able to detect 4T1 breast cancer xenografts by FL-PA imaging, presumably enhancing uptake by tumor lesions through the EPR effect, and to exert a boosted therapeutic effect after laser irradiation compared to CHT alone. A relevant concern of phototheranostic nanoagents is represented by their effective delivery in tumor microenvironment. Very recently, Li and colleagues [97] have tested a biomimetic strategy of camouflage, by coating SPNs with the cell membranes of activated fibroblasts (AF). Such AF-SPNs have proven to be able for cancer-associated fibroblasts targeting after systemic administration, providing not only FL-PA signals for successful detection of xenograft 4T1 breast tumors but also promoting a combined PDT/PTT approach, leading to significant antitumor response. Further efforts are needed in future to overcome challenges like long-term safety or fast renal clearance, biocompatibility, stability, and tumor targeting, to expand the SPNs applications in biomedical research from preclinical to clinical field [97].

## 6. Perspectives and Conclusion

Over the last decade, PAI has attracted growing interest in oncology as it potentially offers relatively low cost, real time, and noninvasive in vivo imaging of tumor lesions complementarily to morphological B mode images provided by US. Critical points to guarantee a successful clinical translation of PAI are the demonstration of its feasibility, safety, and effectiveness. Preliminary preclinical studies have exploited mainly endogenous chromophores such as melanin, HbR, and HbO_2_ to characterize tumor microenvironment, in order to improve diagnosis and therapeutic outcomes assessment. More recently, cancer nanomedicine has rapidly evolved until to design novel PA nanoplatforms, characterized by high biocompatibility and photostability, as well as excellent absorption coefficient extended in the NIR II region, enabling deep tissue analysis with good SNR. Further efforts allowed to functionalize these nanosystems with a large number of targeting moieties and to carry simultaneously multimodal imaging probes and therapeutic agents inside tumor lesions through a single multifunctional tool. However, PAI translation for cancer detection and characterization in clinic is still in infancy, and there are still shortcomings to overcome.

Even if PAI technology has reached preliminary applications in clinical studies, improvements are needed relatively to spatial resolution for visualization of smaller lesions in deeper tissues and temporal resolution for dynamic processes imaging [134]. Recent advances in laser technology, improving the pulse repetition rate and the optical penetration, have been proved highly effective to enhance PAI systems performances for oncological applications, allowing for in vivo monitoring of the metastatic process in a mouse model of melanoma and deep brain imaging in rodents [135]. Further engineering developments with regards to laser sources, detectors, data acquisition, and image processing algorithms of PAI systems will open new perspectives for cancer disease detection and targeted therapy, improving safety regarding laser exposure for patients and operators, as well as SNR and motion artifacts. To enhance PA image contrast, new exogenous molecular imaging agents are under investigation regarding their ability to produce an adequate PAI signal with a relatively low injected dose, avoiding toxic effects, and will need FDA approval for human use.

In addition, limitations in their molecular structure have prompted for developing innovative strategies for a more effective drug delivery, and very recently, innovative smart activatable theranostic nanoplatforms have been tested into a glioma mouse model [136].

Similarly, progresses in the PA instrumentation will be a key factor for the approval regarding clinical adoption and marketing of PAI devices by regulatory committees such as FDA in the United States and CE Mark in European Union. Very recently, a pilot study on healthy patients has proved that PAI offers a good reproducibility for soft tissues characterization, encouraging PAI integration in clinical setting [137].

## Figures and Tables

**Table 1:** Summary of main endogenous and exogenous PA contrast agents.

Imaging agent	Absorption wavelength (nm)	Cancer biomarker	Cancer type	Multimodality	Theranostics	Application	Reference
*Endogenous optical compounds*							
Hemoglobin	760–850	Angiogenesis	Brain, breast, Prostate	—	—	Preclinical/clinical	[7–9]
Melanin	700	*α*v*β*3, acidic microenvironment	Skin, ocular, lung	PET-MRI-PAI	PTT	Preclinical/clinical	[10–22]

*Exogenous optical compounds*							
Indocyanine green	600–900	*α*v*β*3, albumin, EGFR, B7-H3R, UPAR, acidic microenvironment, FR	Lung, liver, colon, brain	MRI-PAI	PTT	Preclinical/clinical	[23–44]
Gold NPs	650–110	*α*v*β*3, acidic microenvironment, FR, siRNA gene-silencing, MAGE, intratumoral temperature, CXCR4	Lung, prostate, ovaries, stomach, skin, brain	CT-MRI-PAI	PTT/PDT	Preclinical	[45–55]
Palladium NPs	826–1068	*α*v*β*3	Breast	—	PTT	Preclinical	[56]
Copper sulfide NPs	826–1068	*α*v*β*3	Breast	—	PTT	Preclinical	[57–61]
Bismuth NPs	1000–1700	EPR effect	Breast, brain	CT-PAI	PTT	Preclinical	[62–64]
Titanium NPs	808	EPR effect	Breast, cervix	—	PTT/PDT	Preclinical	[65–67]
Iron oxide NPs	600–900	FR	Breast, ovaries	PET-MRI-PAI	PTT	Preclinical	[68–70]
Superparamagnetic iron oxide NPs	500–780	HER2	Breast	—	PTT	Preclinical	[71]
Carbon-based NPs	350–700	*α*v*β*3, EGFR	Breast, brain, skin, lung, stomach	FL-PAI	PTT/PDT	Preclinical	[72–84]
Graphdiyne	NPs	690	FR	Breast	FL-PAI	Preclinical	[85]
Semiconducting polymer NPs	660–748	*α*v*β*3, FR	Breast, brain, cervix	FL-PAI	PTT/PDT	Preclinical	[86–97]
